# Improved Folch Method for Liver-Fat Quantification

**DOI:** 10.3389/fvets.2020.594853

**Published:** 2021-01-12

**Authors:** Ramgopal Mopuri, Mugagga Kalyesubula, Alexander Rosov, Nir Edery, Uzi Moallem, Hay Dvir

**Affiliations:** ^1^Volcani Center—Agricultural Research Organization, Institute of Animal Science, Rishon LeZion, Israel; ^2^Department of Animal Science, The Hebrew University of Jerusalem, Rehovot, Israel; ^3^Pathology Laboratory, Kimron Veterinary Institute, Veterinary Services, Rishon LeZion, Israel

**Keywords:** fatty liver, pregnancy toxemia, triglycerides, hepatic fat quantification, lipids extraction, steatosis

## Abstract

Fatty liver represents a significant metabolic pathology of excess intrahepatic fat in domestic animals and humans. Quantification of hepatic-fat content is therefore essential for diagnosis and investigation of liver and metabolic disease. However, the reproducibility of hepatic steatosis analysis is often low due to subjective and technical factors. We hypothesized that improvement in tissue-lipids extraction efficiency would contribute to the accuracy and precision of liver-fat determination. To test it, we investigated the effect of standardized tissue sonication on liver-fat quantification by the Folch method in sheep. Liver samples from grownup lambs of lean (*n* = 16) and fatty (*n* = 15) livers, and from pregnant ewes (*n* = 6) who died from pregnancy toxemia (PT), were used for hepatic-fat content determination with or without tissue sonication. In the grown lambs, an average hepatic-fat content of 6.6% was determined in sonicated compared to 5.1% in non-sonicated specimens (*P* = 0.0002). Similarly, in ewes with PT, an average of 12.5% was determined with sonication compared to 10.8% without it (*P* = 0.0006), and the reproducibility was higher with sonication (CV of 3.1 vs. 6.1%, respectively). Thus, tissue sonication improved the efficiency of liver-lipids extraction and was significant to the accuracy and precision of hepatic-fat determination. Enzymatic quantification of triglycerides was moderately correlated with the results obtained gravimetrically (*r* = 0.632, *P* < 0.005). The reported data provide reliable reference values for pregnancy toxemic sheep. The significant improvement in liver-fat quantification observed with the reported revised protocol is likely applicable to most mammals and humans.

## Introduction

Fatty liver represents an abnormal metabolic condition of excess intrahepatic fat (> 5%, w/w), referred to as hepatic steatosis. In humans, it is recognized as a result of alcohol abuse or viral hepatitis, but most commonly due to overnutrition and sedentary lifestyle (1). Hepatic steatosis may also develop as a result of negative energy balance, as observed in women with pregnancy starvation ketoacidosis (2), in overweight cats undergoing periods of anorexia with little or no food intake (3), and in high-yielding periparturient ruminants (4, 5). Although advances in energetic feeding systems of transitioning ruminants helped in reducing the prevalence of energy-deficiency disorders (6, 7), the physiological energy demands of prolific sheep and goats are often still too high to be suitably met by nutrition (8, 9). In such cases, the induced massive adipose lipolysis resulting in increased liver uptake of circulating fatty acids may exceed the hepatic capacity to oxidize them. Consequently, more of the fatty acid influx to the liver is diverted to the synthesis of ketone bodies and triglycerides, the latter of which can accumulate as intrahepatic fat in hepatocytes lipid droplets (10). Indeed, fatty liver and ketoacidosis are the primary metabolic pathologies of pregnancy toxemia (PT), which is a common and often lethal metabolic disorder of energy deficiency in prolific sheep and goats (11). Accordingly, fatty infiltration to the liver is the most striking post-mortem feature in affected ewes, which is thus used to confirm PT diagnosis made based on the history and clinical symptoms.

The extent of hepatic steatosis can be assessed by histopathology (12), biochemically using either enzymatic (13), or gravimetrical methods (14), as well as by non-invasive imaging techniques (15). However, subjective and technical factors limit both the reproducibility and the extent of agreement between the results obtained by different methods (16).

Several solvent-extraction methods have been employed for tissue-lipids isolation and quantification (17). The use of chloroform-methanol (Chl-Met) as an extraction media (18, 19) has been extensively utilized and used as a standard reference for evaluating hepatic fat by MRI and histology (16, 20). The Folch method (19), which utilizes Chl-Met at a 2:1 (v/v) ratio, has been widely used for the extraction of lipids from animals, plants, and bacterial sources (17, 21). Effective lipid extraction depends on efficient tissue homogenization—*i.e.*, disruption of tissue and cells for particle-size reduction and uniform distribution in the extraction liquid. Whereas, mechanical rotor-homogenization has been a dominant methodology (19, 22), the application of sound energy to disturb particles in solution (sonication) has also been utilized for lipids extraction (23, 24).

Here, we hypothesized that efficient sample sonication would improve the extraction of lipids from the liver tissue and, consequently, the fat-content quantification accuracy. To test it, we have used the Folch method, with and without tissue sonication, in a comparative analysis of the intrahepatic-fat content. The ovine livers extracted post-mortem from grownup lambs (25) and from pregnant ewes presenting with clinical PT permitted precise quantification and sensitive analyses of the differences in extraction efficiencies between the two protocols across a wide range of liver-fat contents. The reported data for the pregnant ewes offer a reliable reference of intrahepatic fat content values in PT. Since the liver tissue structure and cellular anatomy is conserved in vertebrates (26), the results obtained here in ovine, across a wide range of lean and steatotic livers, are likely applicable to all mammals.

## Materials and Methods

### Animals and Experimental Design

All sheep were grown and maintained at the Volcani experimental farm in Rishon LeZion, Israel. The livers were harvested from ram lambs (*n* = 30), which were raised on either *ad libitum* forage-based or concentrate-based rations to, respectively, induce lean or fatty livers for a previous study (25), and from pregnant ewes who died from severe PT (*n* = 6). All livers were harvested immediately after death (pregnant ewes) or slaughter (lambs at ~7 months of age), and ~200 g of the left lobe of each liver was stored at −20°C until further biochemical analysis.

### Determination of Hepatic-Fat Content

Triplicates of ~1 g of tissue were sampled from dispersed zones of the frozen left lobe. The exact wet weight of each sample was determined after thawing and dehydrating the excess moisture on a Whatman filter paper for 10 min at 25°C. The total fat was extracted from the liver samples following the Folch method (19), with a slight modification. Each sample (~ 1 g tissue) was mechanically homogenized in 25 mL Chl-Met (2:1) solution for 2 min, using a rotor homogenizer (HOG-020, MRC ltd, Holon, Israel). One-half of each homogenate was subjected to sonication (VCX 750, Sonics and Materials Inc., Newtown, CT, USA) for 5 min at an amplitude of 30%, with cycles of 5 s on and 5 s off. The second half was not sonicated. Sonicated and non-sonicated samples were then agitated overnight at 25°C, afterward centrifuged at 3000 × *g* for 10 min to collect the supernatant. For removal of polar lipids, 4 mL of 0.9% NaCl were added to the supernatant, and the mixture was briefly vortexed, then centrifuged at 2500 × *g* for 10 min. The upper phase was discarded, and the residual interface was further rinsed twice with 4 mL of 50% methanol. The lower chloroform phase containing the fat (triglycerides and cholesteryl esters) was collected and evaporated in a rotary evaporator under vacuum. The remaining fatty phase was oven-dried at 45°C for 2.5 h to remove residual moisture. The fat weight was determined, and the hepatic-fat content was computed as the percentage of the wet liver weight.

Non-sonicated liver homogenates from 26 grownup lambs were also employed for enzymatic triglycerides determination using the Triglyceride Quantification Kit (ab65336, Abcam; Cambridge, UK) following the manufacturer's instructions.

### Histological Analysis

Fresh liver slices from ewes with PT were fixed in 10% formalin-−100 ml 40% formaldehyde, 900 ml H_2_O, 4 g/L NaH_2_PO_4_ (monobasic) 6.5 g/L Na_2_HPO_4_ (dibasic/anhydrous). Before embedding in paraffin blocks, the formalin-fixed liver samples were dehydrated in a series of ethanol and xylene solutions of increasing concentrations. Thin 5 μm liver slices made using the Microm HM355S microtome (Thermo Fisher Scientific, USA) were fixed onto glass slides, then rehydrated with ethanol followed by periodic acid-Schiff (PAS) staining. Stained sections were mounted in a xylene based mounting media (Tissue-Tek Glas mounting Media, Sakura, Netherlands), and images were captured using a Leica ICC50 digital camera mounted on a Leica DM 500 microscope (Leica Microsystems, Wetzlar, Germany).

### Statistical Analysis

Average values of independently measured triplicates were used for the analysis. Data from the lambs or the pregnant ewes were not significantly deviated from normality, as indicated by the Shapiro-Wilk test. Thus, parametric testing of pairs of sonicated vs. non-sonicated samples was performed via paired student's *T-*test in JMP (version 14.0.0, Cary, NC, USA). Significance was accepted at *P* < 0.05. The Pearson correlation between the results obtained by the enzymatic triglyceride assay and the fat-content gravimetrical determination was calculated using JMP. The intra-assay coefficient of variation (CV) values were calculated based on the triplicates data of each PT animal. Unless otherwise stated, data are presented as mean ± standard error of the mean (SEM).

## Results

**Figure 1:**
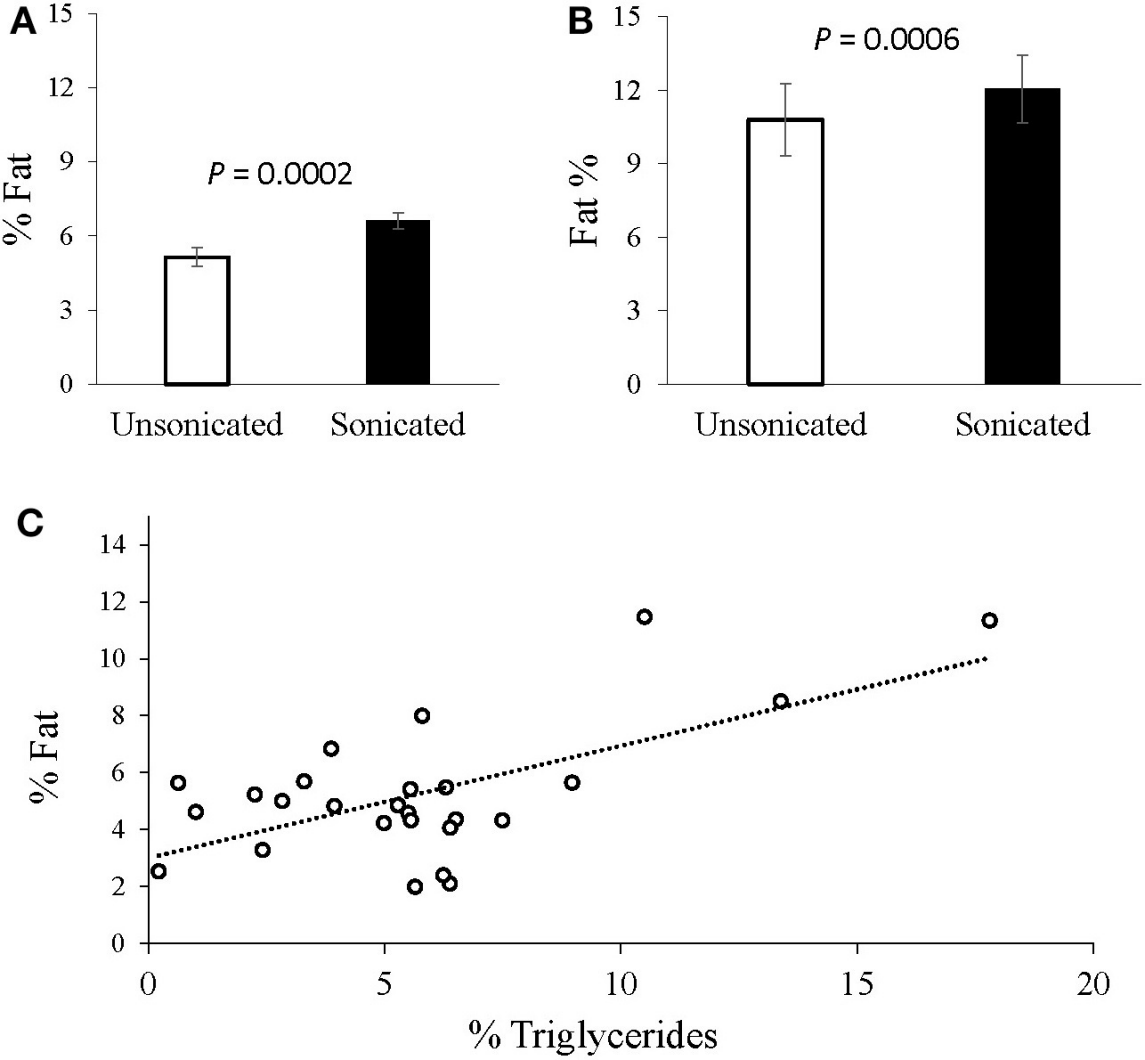
Intrahepatic-fat content analysis with vs. without tissue sonication. **(A)** Livers extracted from grown male lambs (*n* = 30), exhibited greater fat content with sonication applied (6.6 vs. 5.1%; *P* = 0.0002, SEM = 0.35). **(B)** Livers extracted from Pregnancy toxemic sheep (*n* = 6), similarly showed greater fat content with sonication (12.1 vs. 10.8%; *P* = 0.0006, SEM = 0.94). **(C)** Plot of the gravimetrical hepatic-fat contents vs. the triglycerides concentrations determined enzymatically. A moderate overall Pearson correlation was observed (*r* = 0.6317; *P* = 0.005).

In the grown lambs (*n* = 30), sonicated samples yielded higher hepatic fat content values (averaged at 6.6% compared to 5.1% in the non-sonicated; *P* = 0.0002; Figure 1A). Likewise, in the pregnancy toxemic ewes, the measured hepatic-fat content was higher when sonication was applied (12.1 vs. 10.8%, respectively; *P* = 0.0006; Figure 1B). The average intra-assay CV for liver fat extracted from ewes with PT was 3.1% with sonication vs. 6.1% without it.

In grown lambs, the average hepatic fat content determined by enzymatic quantification of triglycerides concentrations was 5.7% ± 3.8 (*n* = 26), whereas 5.1% ± 2.3 (*n* = 30) was obtained gravimetrically. A moderate correlation was found between the two methods (*r* = 0.632, *P* = 0.005; Figure 1C). Further analyses revealed higher positive correlation (*r* = 0.757, *P* = 0.018) in animals having high intrahepatic fat (≥ 5.5% *n* = 9), but no correlation (*r* = –0.078, *P* = 0.7662) in those presented with low intrahepatic fat (< 5.5%, *n* = 17).

**Figure 2:**
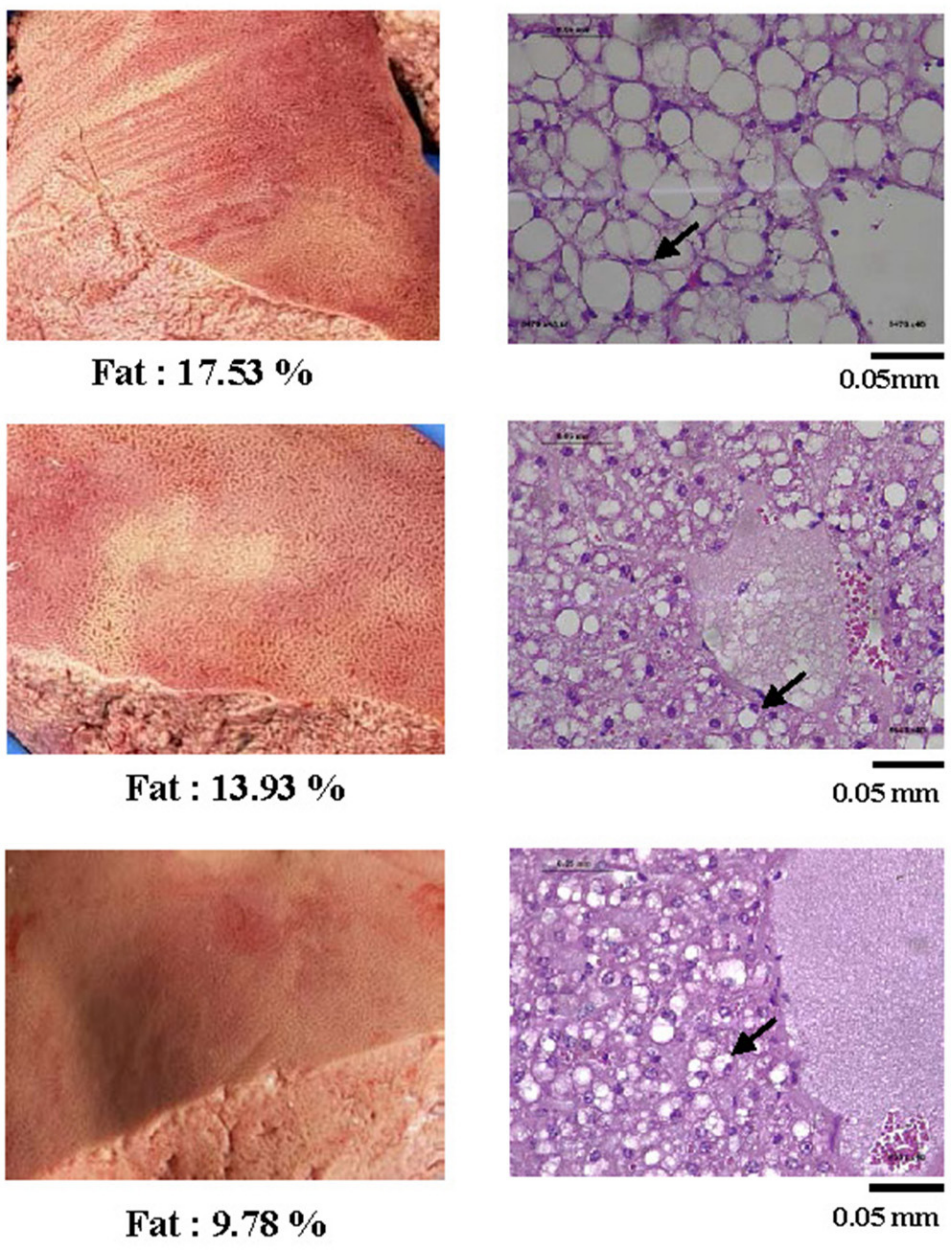
Representative pictures of livers from sheep with pregnancy toxemia (left) and corresponding histological photographs (right) of tissue sections stained by PAS at a 400X magnification. All livers of PT-affected ewes were yellowish and of fragile texture. The histopathology analysis revealed extensive micro and macrovesicular steatosis. Arrows exemplify nuclei pushed aside by large hepatocyte lipid droplets.

In ewes with fatal PT (*n* = 6), the hepatic fat content ranged from 9.5 to 17.5%. These high levels of intrahepatic fat content were consistent with histopathological analyses showing extensive hepatocytes macrovesicular steatosis (Figure 2).

## Discussion

Quantitative analysis of intrahepatic fat is central to diagnosis and severity assessment of fatty liver disease. It is also an essential readout for studying the pathophysiology of fatty liver and evaluating potential modulators of hepatic steatosis. The Folch method is one of the most commonly used biochemical procedures for tissue-fat determination. In the present study, we used freshly-harvested ovine livers to investigate the effect of tissue sonication on fat extraction and quantification efficiency.

Whereas, previous work with bovine livers could not detect a significant effect of sonication on fat extraction (27), our data clearly shows an improvement in fat recovery from all ovine liver samples employed. Overall, the fat-content results were 29.4% higher with sonication in the grown lambs and 12% higher in PT ewes. The capacity of the current study to detect these differences may be attributed to the increase in the sonication dose time (5 min in this study vs. 30 s in the previous study), and possibly to differences in the sample mass utilized (1 vs. 0.25 g, respectively). As can be expected, the improved fat-extraction efficiency with sonication also increased the quantification precision (intra-assay CV of 6.1% with non-sonicated vs. 3.1% with sonicated samples).

The correlation between the Folch method and the enzymatic quantification of hepatic triglycerides concentrations was moderate (*r* = 0.632). This may be partially attributed to the inherent difference between the measured substance; *i.e.*, unlike the enzymatic triglyceride assay, the gravimetrical approach accounts for both the triglyceride and cholesteryl-ester constituents of the fat. Therefore, variation in the proportions of these constituents would compromise the correlation between the results obtained by the two methods.

Inherent technical differences between the methods may also contribute to the observed moderate correlation. For instance, the gravimetric Folch method involves a few unique error-prone steps of organic solvent extraction and phase separation. On the other hand, whereas by signal amplification enzymatic methods offer enhanced detection sensitivity, they also involve additional unique processing steps such as preparation of assay mixtures and blanks that can contribute to the propagation of systematic errors. Moreover, compared to the direct gravimetric method, which provides absolute measurement, indirect enzymatic methods are relative, *i.e*., based on a comparison to standards. As the relationship between the measured signal and the substrate concentrations may vary with concentration, the relative methods are dependent a good mathematical description of this relationship. Additional unique sources of error of enzymatic methods include factors affecting enzyme stability or activity, such as environmental or endogenous inhibitors. Together, these different sources of error between the methods may differentially affect the systematic error.

Interestingly, among the more fatty samples (> 5% intrahepatic fat) the correlation between the two methods was higher (*r* = 0.757; *P* = 0.018), but it was poor (–*r* = 0.078; *P* = 0.76) among the lean livers (< 5%). Whereas, this difference may be partly related to the higher relative measurement errors expected at low-fat levels, it may also indicate a rise in the proportion of the triglycerides compared to the cholesteryl-esters as the hepatic fat content increases.

These findings are consistent with those obtained in dairy cows, in which a high correlation (*r* = 0.8) between total lipids and triglycerides concentrations was observed in a herd with a high incidence of fatty liver, but moderate correlation (*r* = 0.59) in a herd with low incidence (28). Likewise, in alloxan-diabetic and pregnancy-toxemic sheep, characterized by high hepatic-lipids contents, the proportions of triglycerides in the total lipids were 64 and 71%, respectively; however, in normal sheep, it was substantially lower (~7%) (29).

## Conclusions

Application of tissue sonication significantly improved fat extraction from liver samples homogenized by the Folch method. Accordingly, the accuracy and precision of quantitative hepatic-fat content analysis were considerably enhanced. Incorporation of suitable tissue sonication as described here into standard intrahepatic-fat content determinations may advance our ability to diagnose and investigate fatty liver disease in various mammalian species. The reported values can serve as a reference for future studies of fatty liver disease in sheep with PT.

## Data Availability Statement

The original contributions presented in the study are included in the article/supplementary material, further inquiries can be directed to the corresponding author/s.

## Ethics Statement

The animal study was reviewed and approved by Volcani Center Animal Care Committee (permit # 764/18 IL).

## Author Contributions

RM and MK performed the lipids extraction, quantification, analysis, and participated in the paper writing. AR assisted with animal care and liver extractions. NE helped with histopathology analysis. UM provided writing suggestions. HD conceptualized, designed, supervised the experiments, and wrote the paper. All authors contributed to the article and approved the submitted version.

## Conflict of Interest

The authors declare that the research was conducted in the absence of any commercial or financial relationships that could be construed as a potential conflict of interest.

## Abbreviations

PT: pregnancy toxemia
Chl-Met: chloroform-methanol
CV: coefficient of variation
SEM: standard error of the mean.
